# A proposal to make the pulse oximetry as omnipresent as thermometry in public health care systems

**DOI:** 10.7189/jogh.10.0203102

**Published:** 2020-12

**Authors:** Arvinder Singh, Sushila Kataria, Payal Das, Amit Sharma

**Affiliations:** 1 Max Super Specialty Hospital, I.P. Extension, Patparganj, New Delhi, India; 2 Internal Medicine, Medanta Hospital, Gurugram, Haryana, India; 3 Department of Zoology, University of Delhi, New Delhi, India; 4 International Centre for Genetic Engineering and Biotechnology, Aruna Asaf Ali Marg, New Delhi, India

## MONITORING OXYGEN LEVELS

Pulse oximetry is a ubiquitous investigative technique in modern medicine and is commonly used in clinical settings since its advent in 1970s [1]. Pulse oximetry is a non-invasive and painless test that measures arterial oxygen saturation levels that indicate the percentage of hemoglobin binding sites occupied by oxygen which in turn is a function of the arterial PO_2_ as defined by the hemoglobin-oxygen dissociation curve [2]. Pulse oximeters can be of three types with distinct uses: (1) those with finger probe for individual personal use, (2) handheld units which focus just on oxygen saturation, and (3) stand-alone units which incorporate other parameters including ECG, capnography or blood pressure monitoring [3]. Given their small size and affordable prices (US$ 20-50), finger probe oximeters are as handy as thermometers. They can be used repeatedly by patients after adequate sanitization and hygiene. Clear gloves or finger sleeve can also be used by the patients to minimize direct contact with the oximeter in these COVID-19 times [4].

## USES IN ROUTINE MEDICAL PRACTICE

Pulse oximetry has an established role in critical care, and during the administration of general anaesthesia so as to warn clinicians of hypoxemia. The introduction of oximetry as a continuous monitoring device in anaesthetized patients had led to reduction in complications in intensive care unit [5]. Early detection of hypoxemia had also reduced the incidence of myocardial ischaemia in anaesthetized patients [5]. In a large randomized trial with 20 802 peri-operative patients in 1993, hypoxemia could be detected in 7.9% patients using a pulse oximeter vs detection in only 0.4% patients without one. Thus a ~ 19 fold increase in the detection of hypoxemia was noted in the oximeter group than in the control group. It was also observed that myocardial ischemia was more common in the control group vs the oximetry group [6]. In addition, these devices are also useful in monitoring respiratory and non-respiratory diseases like asthma, chronic obstructive pulmonary disease (COPD) [7], congenital heart defects, congestive heart failure, cystic fibrosis, interstitial lung diseases, lung cancer, obstructive sleep apnoea and pneumonia. Pulse oximetry is also used in the management of oxygen levels after events such as drowning, poisoning and allergic reactions. Thus, oximeters have become indispensable in hospital settings.

## COVID-19 AND SILENT HYPOXIA

Pulse oximetry can become an integral component of COVID-19 patients’ respiratory disease management. Most COVID-19 patients do not require hospital admission, however those with acute respiratory distress syndrome may require invasive mechanical ventilation or a respiratory support (in about 5%-15% of COVID-19 patients) [8]. In Asian, African, European and other countries with large populations and moderate to poor health care systems, the hospital settings can be easily inundated with patients seeking respiratory support. Thus, triaging becomes very important. The duration from the initial symptoms of COVID-19 to respiratory failure in most patients is ~ 7 days [9]. Many patients go on to develop “silent hypoxemia”, so-called because of its insidious and hard-to-detect nature. It has been reported that unlike pneumonia due to other infections, COVID-19 pneumonia patients may not feel dyspnoeic or any noticeable discomfort in chest. The physical manifestations become evident when pneumonia has deteriorated to moderate-to-severe levels. The analysis explains the mechanism that air sacs in COVID-19 patients’ lungs do not fill with fluid or pus but they collapse. This reduces the oxygen levels but still maintains the lungs’ normal ability to expel carbon dioxide hence, COVID-19 patients do not feel shortness of breath initially [10]. There is respiratory failure but without signs of respiratory distress, especially in relatively young who were previously healthy or who had only minor underlying conditions [9]. The same has been observed in elderly and with co-morbid conditions as well. However, there are no studies reporting the proportion of COVID-19 patients with silent hypoxemia but there are few case reports that present the clinical scenario in some of the patients [11,12]. Absence of signs of respiratory distress can lead to considerable loss of valuable time and continuous deterioration of patients [13]. In this scenario, oximetry can play a vital role.

## PULSE OXIMETRY AND COVID-19

Temperature, pulse rate, respiratory rate, peak expiratory flow rate and arterial oxygen saturation are considered “Five Vitals” for monitoring a patient with respiratory distress due to COVID-19. Arterial oxygen saturation and respiratory rates can be easily measured in home settings by imparting minimum training to the users. SpO_2_ levels >96% are considered normal, ≥95% are deemed acceptable for home monitoring, and for patients with <93% oxygen, support is indicated in hospital settings [14].

**Figure:**
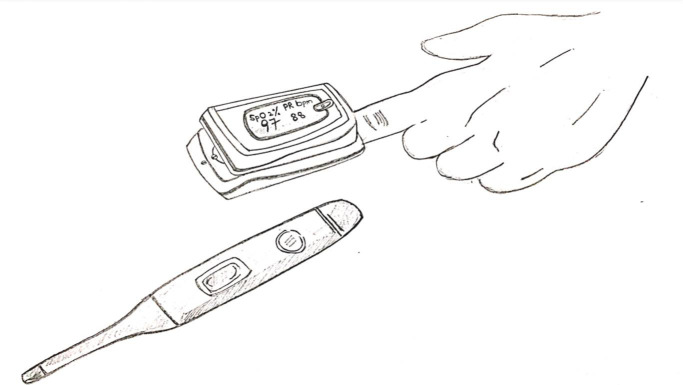
Photo: From the author’s own collection, used with permission.

COVID-19 presents an opportunity to enhance engagement of community with governmental systems on health care issues beyond this pandemic, which itself can learn from previous infectious disease battles [15,16]. Many countries have already adopted oximetry as part of home-based primary and community care measures (United Kingdom, USA, and others). National Health Service (UK) and CDC guidelines recommend use of pulse oximeters in nursing homes, primary care setting and for home care. The Indian government too has recommended use of pulse oximetry in urban settlements as a step towards preparedness and/or containment of excess COVID-19 patients who may otherwise visit hospitals without requirement of urgent care (asymptomatic and mild cases). The guidelines generally specify the levels of SpO_2_ at which medical attention is to be sought. Summary of the guidelines in various countries is given in **Table 1**. Therefore, all countries may consider widespread use of oximetry in COVID-19 patients to assist in triaging [17] as eventually governments would have to resort to strategies to enable home/primary care management of mild COVID-19 cases to ease pressure on hospitals.

**Table 1:** Countries with adoption of pulse oximetry at primary health care/home settings

Country	Purpose	Guidelines
United Kingdom	To monitor and identify ‘silent hypoxia’ and rapid patient deterioration in primary care settings or at home	• Assessment and monitoring of patients in a primary care setting or home
		• Ambulatory patients: assess triaged patients on site
		• Housebound patients
		• Face-to-face or virtual assessment with pulse oximetry +/− rest of observations.
		-Mild: SpO_2_≥95%,
		-Moderate: 93%-94%,
		-Severe: ≤92%
United States. CDC guidelines & Vermont Health Dept.	To allow more rapid detection of clinical deterioration of COVID-19 cases through use of pulse oximeters at nursing homes and home settings	• Within 24 h of RT-PCR positivity, mild cases provided with pulse oximeters.
		• Advice – Seek medical evaluation if saturation falls below 90%
Melbourne, Australia	Assist with anticipated pandemic numbers of COVID patients expected at the hospitals in Melbourne	• Effective monitoring of patients from their homes, allowing for a reduction in people using beds at the hospitals.
		• To be able to safely send patients who do not currently require hospital treatment back home to self-quarantine
		• To rapidly identify a subset of patients whose saturation falls below 92% and need medical attention.
India	Part of active surveillance and response to outbreak by health care workers in urban settlements with inadequate housing and poor conditions	• Health care workers and community volunteers trained in using pulse oximeters in preparedness and response to COVID-19 in urban settlements.
		• Pulse oximeters distributed in quarantine centres and health facilities for self-monitoring/health worker supervision.

## CONCLUSIONS

The early detection of low oxygen saturation levels can forewarn patients and promote prompter medical-attention seeking. The deployment of oximeters would be most beneficial in resource constrained settings where fragile and fatigued health care facilities are evidently unable to deliver medical attention to all COVID-19 patients. As thermometers are to fever management, the pulse oximeter can be to respiratory distress, especially in times of COVID-19.
